# Anaesthetic Management of a Patient With Immune-Mediated Necrotizing Muscle Disease With the Use of a Novel Ultrashort-Acting Benzodiazepine, Remimazolam: A Case Report

**DOI:** 10.7759/cureus.37326

**Published:** 2023-04-09

**Authors:** Hitoshi Ogino, Daisuke Sugiyama, Kenichi Ueda

**Affiliations:** 1 Anesthesiology, Kameda Medical Center, Kamogawa, JPN; 2 Anaesthesiology, Kameda Medical Center, Kamogawa, JPN

**Keywords:** intravenous anesthesia, myopathy, remimazolam, imnm, immune-mediated necrotizing muscle disease

## Abstract

We reported the anesthetic management of remimazolam, a novel ultra-short-acting benzodiazepine, for a 21-month-old female with immune-mediated necrotizing myopathy (IMNM). Remimazolam has a similar chemical structure to midazolam but possesses a unique side chain that reduces its propensity to accumulate in the body, thereby minimizing prolonged sedation or respiratory depression. Our experience supports that remimazolam could be a suitable agent for anesthetizing the patient with IMNM.

## Introduction

Myopathic patients risk severe cardiac and respiratory complications during and after general anesthesia, especially if they have underlying ventilatory muscle weakness and cardiac anomalies associated with muscular dystrophies and related disorders [1]. Volatile anesthetics may induce dysrhythmias through a calcium-channel-related effect, while succinylcholine can induce fatal ventricular arrhythmia by increasing serum potassium levels [1,2]. Anticholinesterase drugs also potentially aggravate autonomic dysregulation [1,2]. Therefore, appropriate anesthetic agents are essential for anesthetic management in patients with myopathies.

Immune-mediated necrotizing muscle disease (IMNM) is an autoimmune myopathy characterized by relatively severe proximal weakness and other symptoms such as dysphagia. Pathological findings include myofiber necrosis with minimal inflammatory cell infiltration. Although the exact prevalence is uncertain, contemporary literature suggests IMNM affects about 7-11 in 100,000 people yearly in the United States [3,4]. The class Ⅱ MHC allele DRB1*11:01 is strongly associated with anti-HMGCR myopathy in adults [3,4]. The presence makes the diagnosis of IMNM of autoantibodies [3,4].

In this context, we report anesthetic management of IMNM using a novel intravenous anesthetic, remimazolam. This drug has a similar structure to midazolam. However, it has a unique side chain that makes it less likely to accumulate in the body, which is advantageous for minimizing the risk of prolonged sedation or respiratory depression. In theory, remimazolam possesses an ideal anesthetic characteristic for patients with IMNM, although further studies are needed to confirm its safety and efficacy in this patient population.

## Case presentation

**Figure 1:**
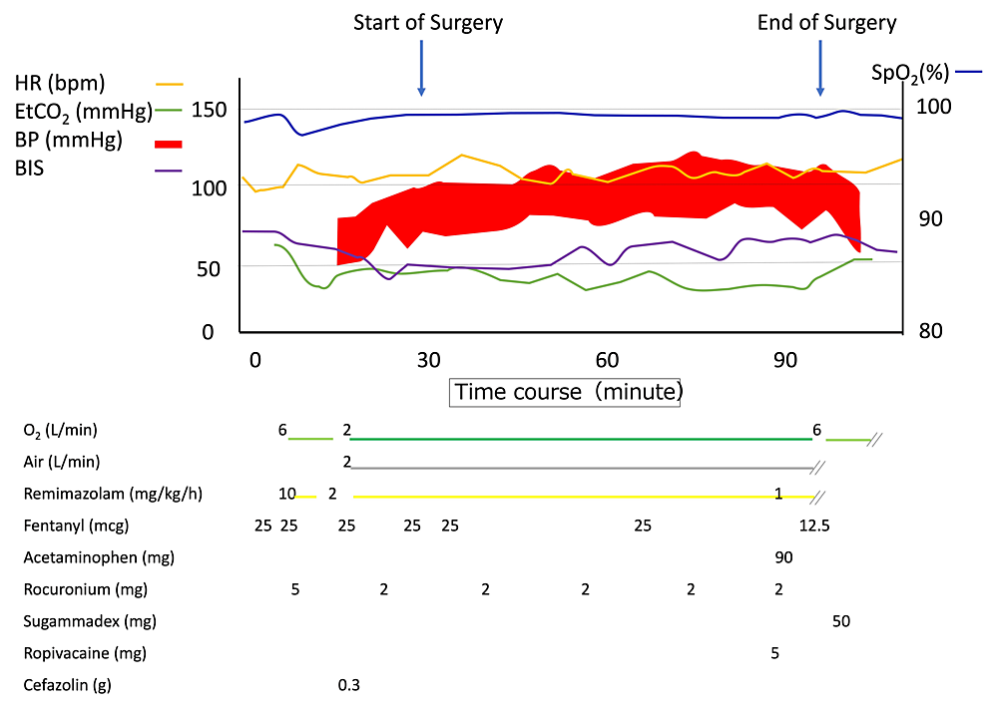
Anaesthetic chart during surgery. The patient's circulatory dynamics were stable during the surgery. After the surgery ended and the administration of remimazolam was discontinued, the patient promptly resumed spontaneous breathing and was extubated and discharged. SpO_2_: Oxygen saturation; HR: Heart rate; EtCO_2_: End-tidal carbon dioxide; BIS: bispectral index​​​​​​​

A 21-month-old female, ASA physical status Ⅱ, 74 cm in height and 8.7 kg in weight, was scheduled for laparoscopic gastrostomy due to failure to thrive. Her delivery was complicated by severe neonatal asphyxia with an Apgar score of 2 and 2 at 1 minute and 5 minutes, respectively, which was later diagnosed as hypoxic-ischemic encephalopathy. She had a history of epilepsy and hypertonia since birth, controlled with antiepileptic drugs (diazepam 3.7 mg/day, sodium valproate 280 mg/day, clobazam 7 mg/day). Due to elevated Creatine Kinase, a thorough examination, including a muscle biopsy, was performed. The results from the biopsy revealed a diagnosis of anti-3-hydroxy-3-methylglutaryl-coreductase (HMGCR) positive IMNM. During the procedure, in addition to ASA standard monitors, radial arterial pressure, BIS®︎ (Nihon-Koden, Japan), and electromyography (EMG) monitor (AF-201P®︎ Nihon-Koden, Japan) were used for intraoperative monitoring. General anesthesia was induced with remimazolam 10 mg/kg/h, fentanyl 3 mcg/kg, and tracheal intubation was facilitated with rocuronium 0.6 mg/kg. After confirming sleep onset by both the BIS® waveform and value (between 40 and 60), the infusion rate of remimazolam was reduced to 1-2 mg/kg/h, 3 mcg/kg of fentanyl was administered as needed depending on the surgical stimulus. During the surgery, 1.22 mg of remimazolam, 160 mcg of fentanyl, and 15 mg of rocuronium were used. At the end of the surgery, 0.375% ropivacaine (total 4 mL) was injected at the incision sites, in addition to intravenous acetaminophen (10 mg/kg) as postoperative analgesia. Spontaneous respiration appeared 5 minutes after discontinuation of remimazolam before sugammadex administration, and the patient was extubated after antagonizing muscle relaxants with sugammadex 4 mg/kg. We were prepared to use flumazenil in this case but did not use it because we determined that the patient's consciousness and breathing were both rapid and sufficient. The operation time was 2 hours and 13 minutes, and the blood loss was 10 mL (Figure 1).

The patient was started on gastric feeding on the first postoperative day. Postoperative laboratory results showed no significant increase in Creatine Kinase (CK) from the baseline, and she did not have a single episode of seizures throughout the perioperative period. The patient was discharged home on the 18th postoperative day without sequela.

## Discussion

IMNM is a relatively new disease concept identified and separated from polymyositis in 2004 [3]. It is a rare autoimmune disease that primarily affects the proximal muscle of the limbs and neck, causing muscle weakness and damage. The most common clinical manifestation is bilateral and symmetrical muscle weakness, which differs from other common forms of myositis. Muscle weakness in the lower extremities often precedes muscle weakness in the upper extremities. However, in IMNM patients, muscle weakness usually starts with the muscle closest to the body's core, such as the hips, thighs, upper arms, shoulders, and neck [3]. IMNM is classified into three types: anti-signal recognition particle (anti-SRP) positive, anti-3-hydroxy-3-methylglutaryl-coA reductase (anti-HMGCR) myositis-specific autoantibodies positive, and seronegative types. The anti-SRP type is characterized by more severe muscle weakness than the other two types, often associated with respiratory insufficiency and worsening prognosis.

On the other hand, the anti-HMGCR type is considered to have milder symptoms than the other two types. However, it is often associated with interstitial lung disease and dysphagia, which poses a risk when administering general anesthesia [4]. The seronegative type of IMNM is associated with malignancy [5]. Clinicians must differentiate IMNM from other forms of myositis, as the treatment approach and prognosis may differ significantly (Table 1).

**Table 1:** Differences between types of IMNM IMNM: immune-mediated necrotizing myopathy; anti-HMGCR: anti-3-hydroxy-3-methylglutaryl-CoA reductase; anti-SRP: anti-signal recognition particle

Types of IMNM	Muscular phenotype	Extramuscular phenotype
Anti-HMGCR-positive	Dysphagia (up to 25%)	No extramuscular manifestations, Cases of heart failure, Likely to be associated with malignancy
Anti-SRP-positive	Dysphagia (30-70%), Muscle atrophy more severe than Anti-HMGCR-positive	Interstitial lung disease Myocarditis
Seronegative		Most likely to be complicated by malignancy among IMNMs

There is limited data on the safety of using various anesthetic agents in this patient population [1]. In this case, the patient was diagnosed as anti-HMGCR positive IMNM, known to have milder symptoms than the anti-SRP type. However, it is unclear whether induction and maintenance of anesthesia with sevoflurane and propofol are safe. Therefore, considering her seizure was well controlled with benzodiazepines and limited data suggests preferable anesthetics for IMNM, she decided to use total intravenous anesthesia with benzodiazepines. Among benzodiazepines, short-acting remimazolam was selected to minimize the risk of postoperative complications such as aspiration pneumonia.

**Figure 2:**
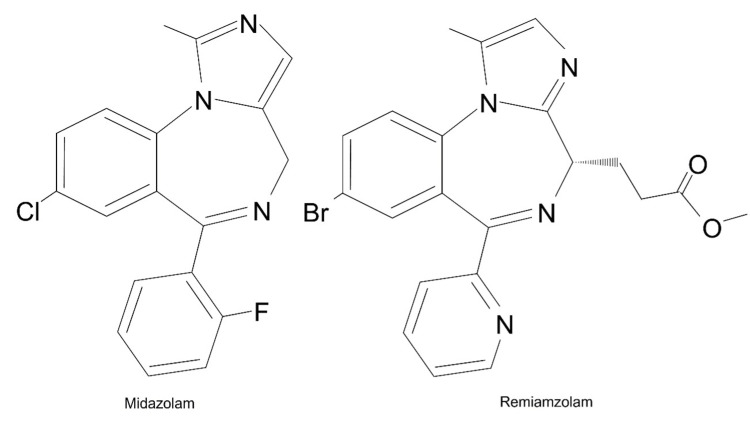
Molecular structures of midazolam and remimazolam. The structure of remimazolam is similar to that of midazolam. The significant difference is that only remimazolam has an ester linkage in its side chain attached to the diazepine ring.

Remimazolam is a unique benzodiazepine that offers several advantages over other intravenous anesthetics. The structure of remimazolam is similar to that of midazolam (Figure 2).

Due to its water-soluble nature and rapid metabolism by tissue esterases to an inactive metabolite, remimazolam has an ultra-short duration of action, which expedites emergence from general anesthesia compared to other benzodiazepines, such as midazolam. Moreover, its pharmacological properties lead to less hemodynamic impact than propofol, which may benefit patients with myopathy at a higher risk for cardiac complications during general anesthesia [6]. Unlike midazolam, remimazolam's inactive metabolite does not have sedative effects. This may be especially beneficial in patients with myopathy, where prolonged sedation can further exacerbate muscle weakness and respiratory complications. Furthermore, remimazolam has a more modulatory effect on the γ-aminobutyric acid A receptor than midazolam, which may result in a more predictable level of sedation and a lower risk of oversedation [7]. Overall, the unique pharmacological properties of remimazolam make it an attractive option for total intravenous anesthesia in patients with myopathy.

## Conclusions

This report presents a case study highlighting the efficacious anesthetic management of a patient afflicted with IMNM. This disease entity has been recently classified as a distinct condition apart from polymyositis. The anesthetic approach adopted in this case involved the administration of a novel intravenous anesthetic agent, remimazolam, an ultra-short-acting benzodiazepine. Remimazolam's administration maintained hemodynamic stability throughout the surgical procedure and ensured rapid emergence from anesthesia, spontaneous breathing resumption, and successful extubation post-surgery. These beneficial pharmacological properties of remimazolam make it an attractive anesthetic agent for patients with myopathy.
